# Bio-Inspired Reactive Approaches for Automated Guided Vehicle Path Planning: A Review

**DOI:** 10.3390/biomimetics11010017

**Published:** 2025-12-30

**Authors:** Shiwei Lin, Jianguo Wang, Xiaoying Kong

**Affiliations:** 1 School of Computer Engineering, Jimei University, Xiamen 361000, China; 2 Faculty of Engineering and Information Technology, University of Technology Sydney, Sydney, NSW 2007, Australia; 3 School of IT and Engineering, Melbourne Institute of Technology, Sydney Campus, Sydney, NSW 2000, Australia

**Keywords:** path planning, automated guided vehicle, artificial intelligence, swarm intelligence

## Abstract

Automated guided vehicle (AGV) path planning aims to obtain an optimal path from the start point to the target point. Path planning methods are generally divided into classical algorithms and reactive algorithms, and this paper focuses on reactive algorithms. Reactive algorithms are classified into swarm intelligence algorithms and artificial intelligence algorithms, and this paper reviews relevant studies from the past six years (2019–2025). This review involves 123 papers: 81 papers are about reactive algorithms, 44 are based on the swarm intelligence algorithm, and 37 are based on artificial intelligence algorithms. The main categories of swarm intelligence algorithms include particle swarm optimization, ant colony optimization, and genetic algorithms. Neural networks, reinforcement learning, and fuzzy logic represent the main trends in artificial intelligence–based algorithms. Among the cited papers, 45.68% achieve online implementations, and 33.33% address multi-AGV systems. Swarm intelligence algorithms are suitable for static or simplified dynamic environments with a low computational complexity and fast convergence, as 79.55% of papers are based on a static environment and 22.73% achieve online path planning. Artificial intelligence algorithms are effective for dealing with dynamic environments, which contribute 72.97% to online implementation and 54.05% to dynamic environments, while they face the challenge of robustness and the sim-to-real problem.

## 1. Introduction

Automated guided vehicles (AGVs) are widely used in logistics, manufacturing systems, port terminals, and industrial automation due to safety, reliability, flexibility, efficiency, and scalability [1,2,3]. The AGV system consists of an embedded controller, vehicle chassis, communication devices, battery, sensors, guidance facilities, and a load transfer device [3]. Path planning obtains a continuous curve that starts from a starting point and ends at a target point, including the defined positions in the path [4]. Optimal path planning attempts to optimize the cost function to optimize the path, considering the time or distance [4].

Optimal path planning can be beneficial for safe and efficient transport in the production process, and it requires low cost and latency, precise positioning and remote control [2,5]. Path planning approaches are classified as online or offline implementation [6]. Online path planning considers dynamic environments or real-time applications, while offline path planning is aimed at static obstacle avoidance. The real-time path planning of AGVs in an unknown environment that still remains a challenge in smart logistics applications [7].

**Figure 1:**
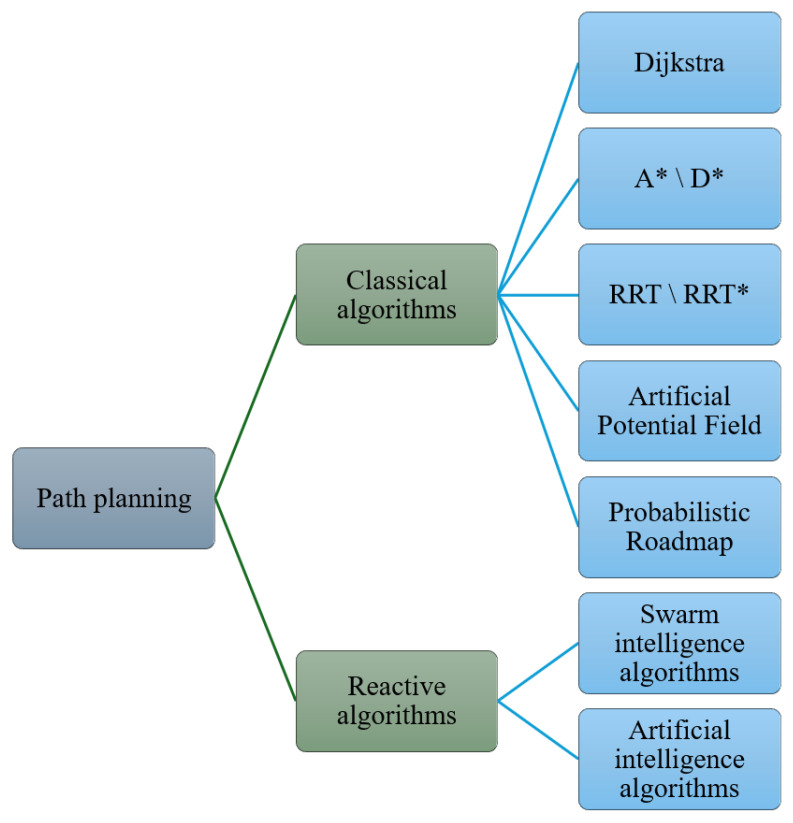
Classifications of path planning algorithms.

The path planning algorithms can be classified as classical algorithms and reactive algorithms, as shown in Figure 1. The classical algorithms include Dijkstra [8,9,10,11,12,13,14], A* [15,16,17,18,19,20], D* [21,22,23,24], Rapidly-exploring random tree (RRT) or RRT* [25,26,27,28,29,30,31,32,33], artificial potential field (APF) [34,35,36,37,38,39], probabilistic roadmap (PRM) [40,41,42,43], etc.

**Figure 2:**
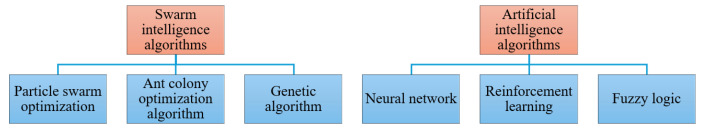
Classification of reactive algorithms.

However, reactive algorithms have drawn attention due to their robust learning capabilities [44]. This review focuses on the reactive algorithms developed over the past six years (2019–2025), including swarm intelligence algorithms and artificial intelligence algorithms, as shown in Figure 2. The swarm intelligence algorithms are inspired by natural behavior to gain the optimal solution based on the fitness functions. Artificial intelligence algorithms, such as neural networks, reinforcement learning, and fuzzy logic, are developed due to their learning abilities and adaptability to dynamic environments.

**Figure 3:**
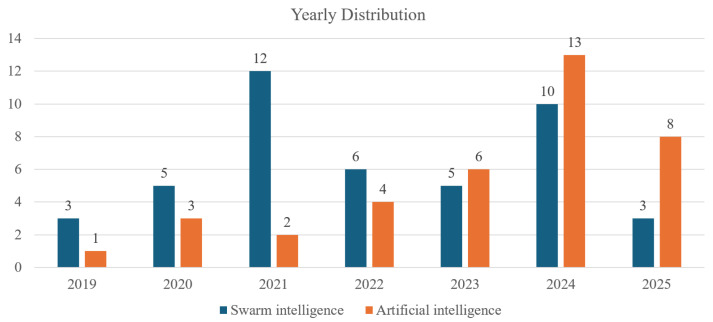
Yearly distribution of the papers.

This review focuses on the reactive path planning algorithms, and the literature was collected through Google Scholar, which supports broad coverage of conference proceedings and journal articles, with the published year restricted to 2019–2015, as shown in Figure 3. The keyword combinations include “AGV path planning”, “AGV reactive path planning algorithms”, “AGV path planning swarm intelligence”, “AGV path planning AI”, and the particular algorithm family, such as “AGV path planning RL”.

Papers are included in the analysis of this review if they implement reactive path planning algorithms, including swarm intelligence algorithms or artificial intelligence algorithms, in the AGV’s scenario or the automated vehicle’s scenario with simulated or experimental validation, and are restricted to the conference proceedings or journal articles. Papers are excluded if they employ classical approaches, address only task assignment problems, or involve scenarios such as underwater vehicles or in-flight drones.

**Figure 4:**
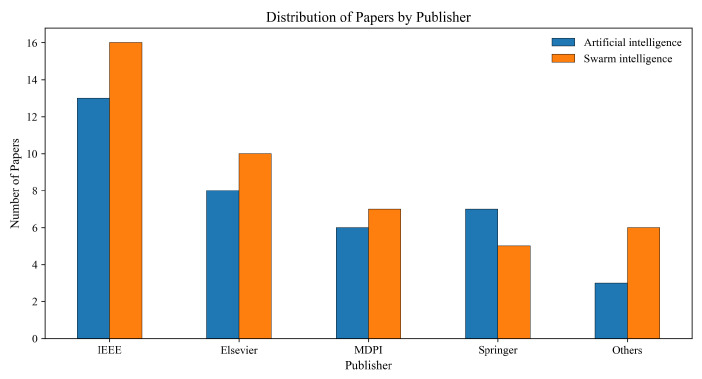
Distribution of the papers by publisher.

This review includes a total of 123 papers, with 81 papers about reactive algorithms and 44 papers based on swarm intelligence algorithms, including 11 papers about particle swarm optimization (PSO), 18 papers about ant colony optimization algorithm (ACO), 9 papers about genetic algorithms (GA), and 6 papers about other swarm intelligence algorithms. For the artificial intelligence algorithms, there are 37 papers, including reinforcement learning (27), neural networks (5), fuzzy logic (4), and other algorithms (1). Most papers are published by IEEE and Elsevier, as shown in Figure 4.

Several survey papers are related to robot or vehicle path planning, such as [6,45]. Lin et al. [6] provides path planning approaches based on the perspective of robots and multi-robot systems, considering the centralized and decentralized decision-making systems and the classification of the algorithms. Reda et al. [45] reviews the models of autonomous driving systems; path planning is treated as part of the autonomous driving systems, and others are about perception, sensors, localization, control, and assessment. However, this paper aims to provide a comprehensive analysis of recent studies, reviewing the research after previous review papers were published (2023–2025). Also, this paper compares the cited studies under the consideration of dynamic environments, online implementation, and multi-AGV coordination, focusing on AGV scenarios.

Section 2 reviews swarm intelligence algorithms, and Section 3 provides the analysis of artificial intelligence algorithms. Section 4 lists the algorithms that are not included in Section 2 and Section 3. This paper compares the algorithms and concludes in Section 5.

## 2. Swarm Intelligence Algorithms

### 2.1. Particle Swarm Optimization (PSO)

The hybrid PSO-SA algorithm is proposed for AGV path planning in the warehouse to avoid the local optimum problem with less time consumption and faster convergence and minimize the path length and obtain a smooth path, which is inspired by the simulated annealing algorithm (SA) [5]. It indicates that a dynamic environment, multiple AGVs, or moving obstacles would be future improvements. Qiuyun et al. [46] introduced an improved PSO for a one-line production line for the shortest transportation time, designing a crossover operation and mutation mechanism to avoid falling into the local optimum, while the scenario is simple, and the algorithm cannot be applied to multiple AGVs.

To balance the performance of exploitation and exploration, Lin et al. [47] presents a hybrid optimization algorithm with probability based on PSO and the cultural algorithm, updating the inertia weight according to the improved Metropolis rule, aiming for multi-AGV path planning. However, it lacks the practical implementation and considerations of dynamics. Gul et al. [48] developed a PSO-GWO optimization algorithm based on PSO and the grey wolf optimizer (GWO), which integrated with a local search technique, considering the path length and smoothness. Although it considers two objectives, the problem formation is not a multi-objective optimization. It cannot be implemented in real-time; the moving goal and multiple robots are considered as future work.

Multi-objective PSO (MOPSO) is combined with the dynamic window approach (DWA) to address the complex environment considering collision avoidance, travel time, and smoothness, while the real environment is not considered, such as environmental uncertainties or real experiments [49]. Zhang et al. [2] presented an energy-efficient path planning algorithm based on the PSO, considering multi-objective optimization, including energy consumption and total execution time. But it lacks considerations of energy consumption data, the transport task execution, or the multi-AGV system.

Ahmad et al. [50] presents a global path planning based on an improved PSO algorithm, which introduces alpha and beta as coefficients to adjust movements and balances safety, time, and distance in path planning. However, it is only implemented in a static simulated environment; path prediction and learning capabilities should be improved. The PSO algorithm is combined with the human learning optimal algorithm to enhance search efficiency and convergence speed, but it is applied only to the single robot system in the static environment [51].

Song [52] presents a global path planning algorithm based on PSO with Levy flight and an inductive steering algorithm, and it considers speed control for safety, while the dynamic situation is a simple scenario. An improved PSO is developed based on ant colony optimization, which presents a collision avoidance factor to optimize the node waiting time and AGV path planning [53] while the situation is offline. The PSO algorithm is utilized to adjust the initial parameters of the ant colony optimization algorithm and investigates the expelling behavior and the elite ant principle for updating the pheromone, while it only provides a simulation of a static environment [54].

Table 1 compares the cited PSO-based algorithms from the perspectives of the properties, considerations, environment modeling, and online operations. Most studies use PSO to optimize path length, smoothness, collision avoidance, or multi-objective optimization with MOPSO. The PSO-based algorithms achieve optimization by updating particles iteratively. The PSO-based algorithms are usually employed for static or simplified dynamic environments (36.36%), and most experiments are achieved with simulation, which reaches 90.91%. The experiments are mostly achieved by simulation with a single-robot scenario, with a proportion of 81.82%, and the main environmental models are represented by the grip map. The percentage of online implementation remains restricted at 36.36%, and the algorithms are suitable for structured manufacturing or warehouse scenarios with predicted obstacles.

### 2.2. Ant Colony Optimization Algorithm (ACO)

An improved ACO algorithm is optimized for a multi-AGV production workshop based on job similarity, multi-objective programming, and a pheromone matrix, which can achieve a faster convergence speed and a shorter maximum time span [55]. It can consider further scenarios, such as flexible job shops or flow shop manufacturing environments in future work. Wang et al. [56] developed an improved ACO for the intelligent parking system with the fallback strategy, valuation function, and the reward/penalty mechanism for the pheromone update strategy, but the efficiency of the algorithm would be reduced if the size of nodes exceeds 1000.

A novel ACO is detailed in [57] by adding a penalty strategy to enhance the exploration of unknown areas with the worst value; however, it lacks a real-world experiment. Modified adaptive ACO employs an improved heuristic function, state transition probability rule, and distribution of initial pheromone concentration to improve the swarm diversity and search efficiency, reducing the path length and turn times [58]. However, the execution time is not competitive when compared with direct search algorithms, and the three-dimensional and multi-objective optimization problems should be paid attention to.

Li et al. [59] introduces grey wolf optimization (GWO) into ACO for improving the pheromone model and adds corner constraints for path smoothness to accelerate the convergence, but it lacks a comparison analysis. Zhou and Huang [60] combines ACO and Dijkstra for the baggage pickup sequencing and AGV path planning for the airport AGV, but it needs to consider multi-AGV conflicts.

ACO is improved with A* multi-directional algorithm to obtain the path, and uses the Markov Decision Process (MDP) trajectory evaluation model to filter and generate the smooth global path in [61]. However, dynamically moving obstacles can be a future direction. Based on the improved ACO and rolling window method, a dynamic path planning algorithm and a second-level safety distance determination rule are proposed in a complex environment [62], while the convergence performance and obstacle avoidance could be further improved.

Wang et al. [63] incorporates fast-scaling RRT* into the ACO algorithm, and it uses dynamic step size strategies, heuristic dynamic sampling, and the two-way search to accelerate speed, but it only focuses on a static environment. Step optimization and path simplification methods are designed to improve the ACO algorithm to avoid low search efficiency, and an adaptive pheromone volatilization coefficient and load balancing strategies are presented for multi-AGV systems [64]. However, it needs to consider conflict resolution in the future.

A hexagonal grid map model is presented in [65], which is used in ACO path planning with a regenerated heuristic factor and a bidirectional search strategy for an intelligent manufacturing system. The future work should concentrate on the robustness of the grid map, search abilities, and real-world experiments. Chen and Yu [66] implements Q-value to adjust the parameters of ACO to enhance algorithm convergence and obstacle avoidance ability, while it is only implemented in static and simple environments and lacks a comparison analysis.

Li et al. [67] designs quantum ACO for optimal and feasible paths based on Bloch coordinates of pheromones and uses a repulsion factor for the space–time distance in an automated container terminal. However, this approach has not been validated in an uncertain environment or real logistics systems. For weak optimization ability and slow convergence of ACO, Ref. [68] employs the fruit fly optimization algorithm (FOA) for pre-searching and the original pheromone distribution anduses ACO for global path planning, but it is only for static, simple environments and lacks comparisons.

To address the problems of path runs and convergence speed, Xiao et al. [69] combines ACO and DWA for indoor AGV global path planning, but it focuses on a static environment and a single AGV. Wu et al. [70] adds the information of the nodes into the heuristic information and the dynamic adjustment factor to guide the information, and introduces the Laplace distribution for the pheromone. However, it is only concerned with static job scheduling.

To improve the optimization effect and search efficiency, non-uniform, and directed distribution of initial pheromones, the adaptive adjustment of iterations and the optimization of parameters by GA are presented to improve the ACO, but it lacks a solid comparison [71]. For operating environments, prior time is introduced, the pheromone increment of the ACO algorithm is modified to minimize running time, and the overall task execution time and distance factors are considered in the pheromone update stage. However, it is not suitable for large-scale or frequently changing tasks [72].

The ACO-based algorithms are compared in Table 2, which usually consider distance, obstacle, and turning angles. The environments can be modeled as a grid or raster map. The ACO-based algorithms face the challenges of limited robustness and difficulty adapting to moving obstacles or changing tasks. The proportions of online implementation and dynamic environments are both 5.56%. The pheromone-based iterations also result in a low rate of multi-AGV scenarios, which reaches only 16.67%. Most cited ACO-based papers are validated through simulations; only 5.56% conduct the experiments.

### 2.3. Genetic Algorithm (GA)

Lyu et al. [73] proposed an integrated scheduling approach with conflict-free path planning based on GA and Dijkstra with a time window, which optimized the number of AGVs, but it does not consider dynamic scheduling and job sequencing problems. Zhong et al. [74] described a conflict-free multi-AGV path planning, which combines GA and PSO with a fuzzy logic controller for efficiency and reliability in automated container terminals, but it took lengthy computation time and cannot support real-time scheduling.

For a logistics system, a three-stage model is designed for task assignment and speed control based on the GA algorithm and simulation, but it lacks comparison with other algorithms or research on AGV charging [75]. A hybrid GA/heuristic is offered in [76] for a cellular manufacturing system to minimize the intercellular movements and the makespan costs in which cell formation problems are designed as a fuzzy mixed-integer linear programming model. Nonetheless, the article only optimizes several problems and is not related to the real case.

Wu et al. [77] considered the blocking of buildings and flight heights when performing the surveillance tasks and outlined a hybrid EDA-GA algorithm for the cooperative path planning, then applied an online local adjustment strategy for the changes of the requirements. The approach may be used for more applications in the future. GA incorporates ACO to improve the initial population, and it considers path smoothness in [78]. It introduces a three-stage mutation operation inspired by the SA algorithm, while it lacks comparison with the SOTA algorithms.

GA is improved for dual-AGVs to ensure efficient and safe actions with a fitness function, and conducted an experiment in leader–follower ROS AGVs [79], but it only focuses on static obstacles. For forklift and latent AGVs, the A* algorithm and the GA are designed as a two-stage optimization model with cyclic rules and a penalty function [80]. However, its scenario only includes static obstacles, and the charging problem is not considered. Farooq et al. [81] presents an improved GA within spinning drawing frames for multi-AGV decision-making and path planning to meet the real-time requirement, which uses time-dependent and time-independent variables as decision variables to minimize the path length, but it is only compared with the traditional GA.

Table 3 presents the GA-based algorithms that implement selection, mutation, and crossover operators to determine the AGV paths. Additionally, 33.33% of the cited GA-based papers achieve online implementation, and 88.89% are based on multi-AGV scenarios. They handle the optimization problem with completion time, makespan-related costs, safe movement, path length, and smoothness. GA-based approaches are also mostly validated by the simulation in a static environment, with 22.22% of both dynamic environments and experimental validation. Moreover, 77.78% of the GA-based algorithms integrate with other classical approaches, swarm intelligence algorithms, or fuzzy logic controllers to achieve better performance.

## 3. Artificial Intelligence Algorithms

### 3.1. Neural Network (NN)

Sung et al. [82] outlined a neural network with offline training and online path planning, which uses the Bellman–Ford algorithm and a quadratic program for offline neural network training in a grid-based graph to minimize the sum of the distances. Despite this, it is hard to acquire ideal situational awareness, and the large size of trained data and the increased dimensionality are the weaknesses.

A recurrent deep neural network with long short-term memory (LSTM) is utilized for the AGV parking maneuver, and it uses an adaptive learning tracking control algorithm for controlling the motions, considering the shortest time, collision avoidance, and the process and terminal costs [83].

For warehouse logistics, Zhang et al. [84] integrates advanced neural networks within the ACO model with a congestion-aware loss function and an adaptive attention mechanism, but the environment model is not clear. Sun et al. [85] combines the A* algorithm and the NAR neural network in the 2D maps, which uses real-time and historical data to establish the NAR neural network, but the success rate is a little low.

Zhang et al. [44] designs a three-layer structure, with the first layer employing the target area adaptive RRT* for data collection, the second layer usning a deep neural network to train the model for learning the relationships between sampling and states, and the third layer using the model to guide RRT* sampling. Its future research focuses on kinematic information, transfer learning, and 3D scenarios.

Table 4 lists the cited model based on the neural network. These models usually combine with the global path planning approach and learn the mapping between the environment and decisions. The NN-based models consider path length, obstacles, or motion planning, and can model the environment as a graph or a grid map. Most models are validated through simulation and can be implemented online. Forty percent of the models consider a dynamic environment, with a single robot scenario.

### 3.2. Reinforcement Learning (RL)

DQN is integrated with a state-dynamic network model to improve the convergence speed in [86], and it uses a distributed training framework for decision-making, while the collaborative navigation still needs to be improved. Yang et al. [87] used the A* algorithm for path planning in a static environment as a priori knowledge, then used the improved Deep-Q network (DQN) algorithm on a semi-known environment to address the problem of excessive randomness and slow convergence, but the improvements can be further based on the local path planning ability.

Xiao et al. [88] designs an improved DQN algorithm based on a dynamic temperature adjustment mechanism and the priority experience replay mechanism and uses a refined multi-objective reward function to guide the path. Sensor noise and dynamic obstacle prediction modules in the real-world experiment should be considered in future research.

Dueling DQN is integrated with prioritized experience replay, which considers position, velocity, and target [89]. It processes multimodal sensing information, but it could consider multi-agent reinforcement learning for simultaneous path planning in the future. A digital twin model is introduced based on an improved dueling double deep Q network (D3QN) at vertical and horizontal levels for resource allocation settings, which implements count-based exploration [90]. Its future work would consider synchronization of manufacturing systems’ activities and multi-resource production scheduling.

Deep reinforcement learning (DRL) and recurrent neural network (RNN) are combined for multi-AGV path planning in [91], employing LSTM and proximal policy optimization (PPO). Although it can deal with sudden failures or temporary changes, the computational time could be further reduced and the model evaluated for dynamic conflict avoidance strategies.

Nie et al. [92] improves the PPO algorithm with sample regularization and adaptive learning rate, which adjusts the action probability density and learning rate to enhance the stability and convergence speed. However, it lacks a real experiment, and its future work would focus on global path planning. The curiosity mechanism is integrated in the PPO method to consider the sparse external rewards and dynamic obstacles, while it cannot guarantee safety in the training [93].

For collaborative multi-AGV systems, Shi et al. [94] presents a framework based on multi-agent PPO and GNN to improve decision-making and local perception, and it uses an RRT-guided mechanism for training. However, it focuses on the simple dynamic simulation environment. The intrinsic curiosity module (ICM) and LSTM are introduced into the PPO algorithm, but obstacle avoidance is effected by the speed of moving obstacles or by obstacle not following regular patterns [95].

Yu et al. [96] uses A* for generating global paths to guide the MAPPO algorithm for solving the problem of deadlock and conflicts, and MAPPO is for local path planning. Its reward function accumulates penalties on movement steps, boundaries, and obstacles collisions, while it is only applied on the single-robot system. Ref. [97] uses accepted–rejected sampling to generate points to be the states of Q-learning, but it lacks the modification of Q-learning.

Q-learning is combined with a Kohonen network, as a Kohonen Q-learning algorithm, and integrates the improved GA into the scheduling policy for global path planning [98]. However, it is only suitable for a simple task scheduling scenario. To improve the efficiency, Guo et al. [99] adds a learning process into the Q-learning algorithm for faster path planning than the traditional Q-learning algorithm, but it only investigates static obstacles.

Gao et al. [100] combines Q-learning and a contract net protocol for multi-AGV dispatching problems, but its comparison analysis is weak, and it only implements the traditional Q-learning method. According to the dynamic real environment, digital twin-driven Q-learning is proposed to solve the path planning problem on production logistics systems, with locations and destinations of all AGVs [101]. However, it is not suitable for complex scenarios.

For Industry 4.0, Hu et al. [102] presents a self-adaptive traffic control model based on Q-learning and behavior trees to prevent collisions at intersections, but it is only suitable for simple simulation circumstances. Huang and Wang [103] employs a beetle antennae search algorithm for initiating the Q table to get rid of the local optimum and introduces a gradual Epsilon–Green algorithm, but it cannot be adapted to dynamic obstacles.

In the shared charging system, a hybrid model to obtain optimal paths, forecast channel flow, and recognize congested regions is introduced in [104] based on ACO and Q-learning and adds a positive ant colony feedback mechanism to maximize efficiency. However, it is only compared with the traditional algorithms in a static environment. Tian and Yang [105] implements a distributed Q-learning for multi-AGV planning. It combines action replanning and map training, considering turning rewards and dynamic priority. However, it only focuses on the simple static environment and lacks considerations of dynamics.

For the intelligent manufacturing workshops, deep Q-learning achieves AGV path planning based on a neighborhood weighted grid modeling method, experience replay pool, and the direction reward function in an unknown environment, but it only concerns the static environment [106]. A path optimization model is presented for the port environment based on the APF and twin-delayed deep deterministic policy gradient (DDPG) framework to guarantee the safety and smoothness of the path, while it cannot deal with the dynamic or real environment [107].

MADDPG is improved with an epsilon-greedy policy to avoid obstacles and minimize energy consumption, which balances exploitation and exploration, but it has not considered optimal values [108]. Guo et al. [109] presents a composite auxiliary reward for a soft actor–critic-based RL model, and it utilizes sum-tree prioritized experience replay for real-time control, but it is only validated in simulation. Wang et al. [110] combines an improved ACO algorithm and the Dnya-Q method to improve execution and path planning efficiency, based on the improved heuristic method, but it does not have a comparison analysis.

Guo et al. [111] presents a pioneering decentralized path planning model to address the scalability limitations of the traditional algorithms. It uses local observations and designs a reward function and state space to avoid collisions. However, the high density of obstacles would affect the algorithm. An improved Dyna-Q method is designed for AGV path planning, and it uses a global path guidance to reduce the path search space [112], but it only compares the model with the traditional reinforcement learning algorithms.

Table 5 summarizes the cited reinforcement learning model. The popular RL-based models are based on Q-learning, PPO, and DQN, and the agents learn the policies by interacting with the environment. Here, 66.67% of the cited papers consider online decision-making, and 55.56% perform in dynamic environments. Additionally, 37.04% of these models could be applied to multi-robot systems, such as a logistics system, and 14.81% are validated by experiments, while the sim-to-real problem remains a challenge.

From Table 5, value-based models, such as Q-learning and DQN, are frequently implemented in low-dimensional or discretized spaces with reasonable sample efficiency, while their scalability to highly dynamic environments is limited. Policy-gradient and actor–critic models, such as PPO and SAC, are the recent trend in RL-based studies. They are suitable for continuous control problems, and most of them can perform online path planning. However, these models are sensitive to the reward design functions; the safety cannot be guaranteed during the training, and the deployment in real-world systems remains a challenge, especially the sim-to-real transfer problem.

### 3.3. Fuzzy Logic (FL)

The elite strategy and the rank-based ant system are utilized to improve ACO and integrate fuzzy logic for dynamic environments such as the FLACO, selecting the optimal path based on travel time and distance, pollutant emissions, and fuel cost [113]. FLACO can be further optimized and extended for a group of vehicles. Considering lane lines, obstacles, and velocities, Ref. [114] presents a hybrid APF-model predictive controller (MPC) based on a fuzzy logic system to adjust the coefficients in the port environments, while precise AGV modeling would be required.

Zhou et al. [115] adopts fuzzy controllers for adjustment coefficients, security, and direction, with an improved ACO and DWA algorithms, and the improved ACO involves a reward and punishment mechanism, but it is a static environment. Ambuj and Machavaram [116] presents a hybrid control strategy based on an improved A* algorithm, which is combined with DWA, which reduces the average path search time, path length, and search grid size. It integrates the PID controller with the adaptive neuro-fuzzy inference system, while it needs to improve the applicability and robustness of the algorithm, and conduct real-world experiments.

Table 6 lists the cited FL model. FL-based approaches integrate with global path planning methods, such as ACO, DWA, GA [74], etc., with 75% of the cited papers considering the dynamic environment, and treating travel cost, velocities, safety, and distance as the objective function. FL-based approaches are evaluated in simulation environments, with online performance to handle uncertainty.

## 4. Others

For AGV sorting systems, Wang et al. [117] proposes an SVM-based model and a temporary target selection algorithm to enhance dynamic path planning, while the model transfer methodology needs to be further developed. BDE-Jaya is presented in [118] for multiple AGVs in a matrix manufacturing workshop to minimize transportation cost, total tardiness, early service penalty with the developed key-task shift, an insertion-based repair method, and three offspring generation methods to improve exploration and exploitation capability. Practical constraints, production environments, multi-objective optimization, and reinforcement learning are treated as future work.

To overcome the local minimum problem, the dynamic enhanced firework algorithm is presented in [119] to enhance the performance of APF, whose optimization objects are path smoothness and safety; incorporating personalized driving style could be a future improvement.

Zhang et al. [120] developed an improved sparrow search algorithm to consider the risk degree, path acquisition time, distance value, and the total rotation angle value based on the linear path strategy, a new neighborhood search strategy, and a multi-index evaluation method. However, it lacks real experimentation, the application of a multi-robot system, and dynamic obstacles. Guo et al. [121] combines GWO and a Kalman filter and uses partially matched crossover mutation operations and an elite strategy for optimization, but it is not validated in dynamic or real-time environments.

An integrated framework is presented in [122] to address AGV path planning, machine scheduling, and process route selection, and it is based on a hybrid variable neighborhood differential evolution (DE) to maximize make span and ensure collision-free operation. However, the AGVs’ speed and multi-objective optimization are not considered. Zhou et al. [123] introduced an artificial fish swarm algorithm for global path planning and applied Markov chain into a trail-based forward search for unforeseen obstacles, using a multiconstrained model predictive controller to calculate command signals, but lacks comparison with state-of-the-art algorithms. The comparison of the above algorithms is listed in Table 7.

## 5. Discussion and Conclusions

### 5.1. Discussion

**Figure 5:**
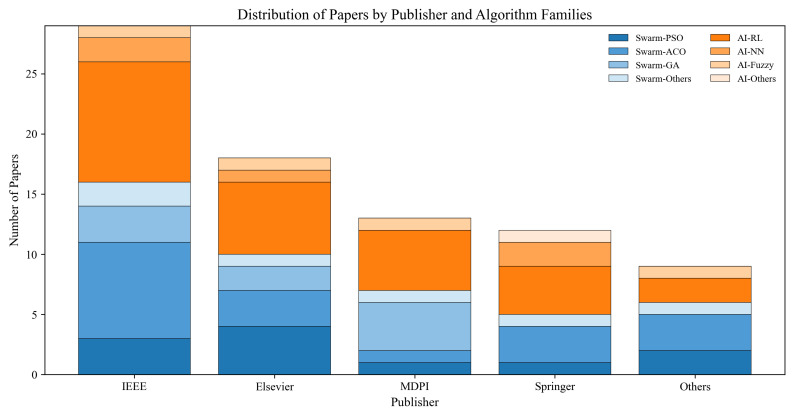
Distribution of papers by publisher across all algorithm families.

**Figure 6:**
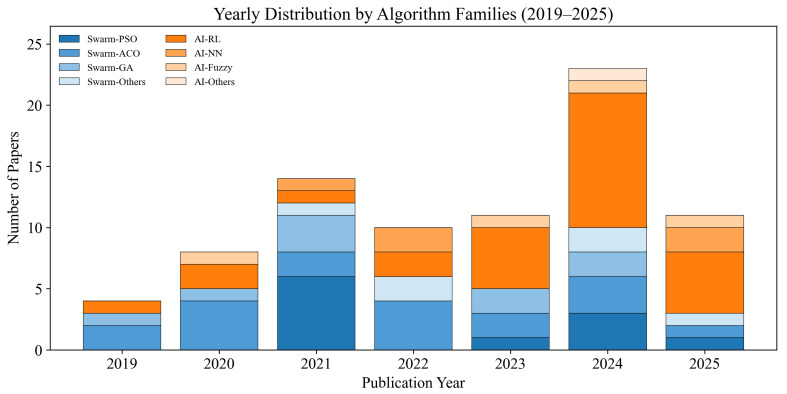
Yearly distribution of the papers across all algorithm families.

This review analyzes the cited reactive AGV path planning algorithms, including swarm intelligence and artificial intelligence algorithms published in 2019–2025. The distribution of the publishers is shown in Figure 5. More precisely, Figure 6 indicates the yearly distribution of the cited papers included per algorithm category, which demonstrates the increasing trend of AI-based approaches.

Compared with the previous survey work [6,45], this review focuses on AGV path planning algorithms, rather than the algorithm classification, decision-making strategies, or system-level review. It provides the perspective from scenario properties, environment settings, experimental validation, and multi-AGV coordination. Table 8 and Table 9 compare swarm intelligence and artificial intelligence algorithms for AGV path planning from the aspect of the papers’ contributions and limitations or future research.

From the literature, PSO, ACO, and GA are the main swarm intelligence algorithms used in AGV path planning. Only 22.73% of the swarm intelligence algorithms in the literature achieve online path planning. Meanwhile, 79.55% of these papers’ environmental properties are based on a static environment, and 34.09% consider the multi-AGV system. Additionally, 65.91% of the algorithms are presented as a hybrid approach, which integrates with other algorithms, such as DWA [49,69,73], SA [5,78], GWO [48,59], A* [61,80], RRT* [63], etc.

The scenarios include manufacturing workshops [2,73,76,118], warehouse [5], production workshops [46,55], airports [60], automated container terminals [67,68], and urban environments [77]. The considerations of AGV path planning achieved by the swarm intelligence algorithms mainly concern path length [5,56,57,60,65], energy consumption [2,49,80], transportation/completion time [46,55,74,75,80], turning times and angles [61,68,69], path smoothness [78,119,121], and obstacle avoidance [66,67,121].

For the AI-based approaches, DQN, PPO, and Q-learning are the most popular models in the cited papers. The percentage of papers that achieve online implementation reaches 72.97%, and 54.05% of properties are in a dynamic environment. However, 32.43% of the cited papers are presented for the multiple AGVs, and 94.59% combine other approaches for better performance, including LSTM [83,91,92,95], ACO [84,104,110,113,115], A* [85,87,116], DWA [115,116], etc.

The implementational scenarios mainly involve intelligent storage systems [87,91], automated terminals [114], logistic systems [89,101], and manufacturing workshops [106]. Distance [82,86,91,92,100], path length [84,87,98,116,117], collision avoidance [44,83,87,95,102,108], process costs [83,90,103,113], motion [85,89,94,105,114], and smoothness [97,107,115,116] are the considerations.

**Figure 7:**
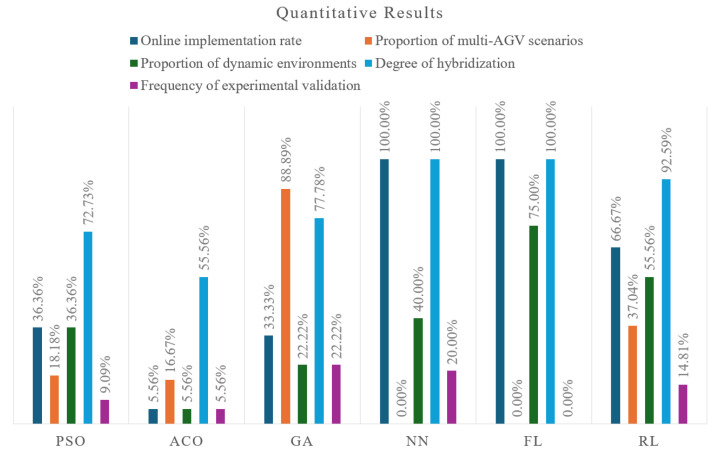
The quantitative results across all algorithm families.

The percentage of papers on AI-based approaches achieving online path planning in dynamic environments is higher than that of swarm intelligence algorithms, while the implementation of multi-AGV systems shows no significant difference. We found that 45.68% of the swarm intelligence and AI-based approaches achieve online implementation, and 33.33% are presented for multi-AGV systems. Figure 7 compares the quantitative results across the algorithm families, including their online implementation rate, proportion of multi-AGV scenarios, proportion of dynamic environments, degree of hybridization, and frequency of experimental validation.

The swarm intelligence algorithms, such as PSO, ACO, and GA, obtain the optimal path for a complex search space by considering problems independently [45]. However, one of the limitations of these algorithms is that they tend to be restricted in the static environment or simple dynamic environment and most of them cannot handle environmental uncertainties or changing environmental conditions [5,49,51], such as dynamic moving obstacles [5,61], dynamic scheduling [70,73,74], AGV conflict resolution [60,64], and moving goals [48].

The PSO-based algorithms enhance path planning ability by combining with other algorithms or introducing new factors, but they face the challenges of environmental uncertainties or a dynamic environment. The PSO-based approaches are suited for static or simplified dynamic AGV planning tasks. The ACO-based methods implement a penalty strategy, the pheromone-guided mechanism, or a search strategy, which results in limited robustness. The GA-based approaches enable global and local search, optimize the multiple AGV scenario, and integrate with some AI-based approaches, but slow convergence and static environments remain limitations. Therefore, they are more suitable for offline optimization.

The AI-based models, such as DQN, PPO, Q-learning, and neural networks, require training data to build the model or learn the policy from the environment [45]. The limitations of these models include hard-to-obtain perfectly trained data [82] or modeling the environment [84,106,107,114], and the training/computational time is long [91,113]. Also, robustness or applicability could not be measured for AI-based approaches [88,116], and they cannot always guarantee safety/completeness [93,108].

The NN-based models implement global path planning methods to enhance efficiency, but their performance relies on the quality of training data, and the unpredictable or new environment model would be a challenge. The RL-based models are popular approaches for the current AGV path planning. They learn policies from the environment and can deal with uncertain environments; however, the sim-to-real transfer and training efficiency are the major problems. The FL-based approaches also combine with the classical algorithms for inference or prediction, while their adaptability and scalability are limited.

From the perspective of the algorithm families, based on the Table 1, Table 2, Table 3, Table 4, Table 5, Table 6 and Table 7, this review could be considered from the application scenario. The cited papers can be classified into four different scenarios, including (1) single AGV path planning, (2) multi-AGV path planning, (3) dynamic environment, and (4) static environment.

From the perspective of the application scenario, swarm intelligence algorithms tend to be applied in a structured environment or without unpredictable moving obstacles, while artificial intelligence algorithms are more applied to a dynamic environment. The Multi-AGV scenario is frequently solved using GA and RL among the cited papers. Figure 7 presents the online implementation rates of the algorithm families, the proportion of multi-AGV scenarios, and the proportion of dynamic environments.

For the AGV path planning, sim-to-real transfer is a significant problem that needs to be considered. The computational load of these reactive algorithms is quite large for onboard computation, and the adaptability to dynamic scenarios is limited [94,101]. Moreover, the dynamics of AGVs are not considered [105], and the problems of sensor noise, bias, and real experiments rise. Most papers use simulation for validation, even though the real experiment is limited to a small scenario, which is hard for real industrial implementation. Transfer learning [44] or model transfer methodology [117] could be considered as a future direction. Also, the embodied intelligence with environmental perception would be helpful for real-time interaction.

### 5.2. Conclusions

From Table 8 and Table 9, AI-based models have become a major trend in current AGV path planning research. Recently, AGV systems have been increasingly deployed in dynamic and uncertain environments, and collaborative AGVs are employed for large-scale tasks. As a result, online capability, scalability, and adaptability are required.

Swarm intelligence algorithms are commonly used approaches in AGV path planning because they demonstrate fast convergence and low computational complexity when generating optimal paths and can effectively optimize objective functions. They are suitable for static or simple dynamic environments; however, their adaptability to environmental changes remains limited.

By contrast, AI-based models, especially reinforcement learning, have recently attracted increasing attention in AGV path planning research. These methods support online path planning, multi-AGV systems, and dynamic obstacle avoidance, as the proportion of online implementations reaches 72.97%, and the percentage of studies considering dynamic environments reaches 54.05%.

From the literature, only 13.58% have real-world experiments for both AI-based and swarm intelligence-based algorithms, as inferred from Table 1, Table 2, Table 3, Table 4, Table 5, Table 6, Table 7, Table 8 and Table 9. Most studies rely on simulated validation, while experiment-driven research or real-world system deployment is lacking. Moreover, 35.80% of the literature aims to enhance adaptability to dynamic environments, while the environment uncertainty is simplified, and robustness or completeness cannot be guaranteed. AI-based approaches face the challenge of safety during training, generalization ability, and the sim-to-real problems.

Motivated by the analysis, some research questions could be considered for future AGV path planning research, as follows:

1. How to reduce the gap between the simulation environment and the real-world AGV operation environment, or how to enhance the realism of the simulation environment when validating the algorithms?
2. How to address environmental uncertainty and unpredictable obstacles when maintaining the online implementation of the algorithms with the safety and completeness constraints of path planning?
3. How to improve the sim-to-real transfer or generalization ability of the AGV path planning algorithm through embodied intelligence, transfer learning, or other approaches?

## Figures and Tables

**Table 1:** Comparison of the PSO-based algorithms.

Paper	Algorithm	Consideration	Model	Online	Properties	Scenario	Hybrid	Experiment
[2]	MOPSO	Multi-objective optimization: energy consumption, total execution time	Graph	No	Dynamic conditions	Single robot, manufacturing workshop	No	Simulation
[49]	MOPSO, DWA	Energy consumption, collisions, travel time, smoothness	Grid	Yes	Dynamic	Single robot	Yes	Simulation
[46]	PSO	Shortest transportation time	-	No	Static	Single robot, one-line production line	No	Simulation
[5]	PSO, SA	Path length and smoothness, collision avoidance	Binary map	Yes	Static	Single robot, warehouse	Yes	Simulation, Experiment
[48]	PSO, GWO	Path length and smoothness	2D map	No	Static	Single robot	Yes	Simulation
[50]	PSO	Safety, time, and distance	2D map	No	Static	Single robot	No	Simulation
[52]	PSO	Smoothness, path length	2D map	Yes	Dynamic	Single robot	Yes	Simulation
[53]	PSO, ACO	Conflict avoidance, total driving time	Node	No	Static	Multi-robots, Workshop Material Distribution System	Yes	Simulation
[47]	PSO, GA	Length, collision	Grid space	Yes	Dynamic	Multi-robots	Yes	Simulation
[54]	PSO, ACO	Length, collision	Grid space	No	Static	Single robot	Yes	Simulation
[51]	PSO, Human optimization algorithm	Convergence, length	Raster map	No	Static	Single robot	Yes	Simulation

**Table 2:** Comparison of the ACO-based algorithms.

Paper	Algorithm	Consideration	Model	Online	Properties	Scenario	Hybrid	Experiment
[55]	ACO	Total completion time, transportation time, time for processing the job	Grid space	No	Static	Multi-robots, production workshop	No	Simulation
[56]	ACO	Path length	Topological map	No	Static	Single robot, AGV-based intelligent parking system	No	Simulation
[57]	ACO	Path length	Grid space	No	Dynamic	Single robot	No	Simulation
[58]	ACO	Path length, turn times	Grid space	No	Static	Single robot	No	Simulation
[60]	ACO, Dijkstra	Path length	Grid space	No	Static	Single robot, airport	Yes	Simulation
[61]	ACO, A* Multi-Directional	Distance, turning times and angle	Grid space	No	Static	Single robot	Yes	Simulation
[62]	ACO, rolling window	Path length, energy consumption	Grid space	Yes	Static	Single robot, complex dynamic environment	Yes	Simulation
[63]	ACO, RRT*	Path length, iterations, runtime	Grid space	No	Static	Single robot	Yes	Simulation
[64]	ACO	Distance	Grid space	No	Static	Multi-robots	No	Simulation
[65]	ACO	Path length	Grid	No	Static	Single robot	Yes	Simulation
[66]	ACO	Iterations, obstacle avoidance, path smoothness	Grid	No	Static	Single robot	Yes	Simulation
[67]	ACO	Distance, obstacle	Grid	No	Static	Single robot, automated container terminal	Yes	Simulation
[68]	ACO	Path length, turning angles	Matrix yard storage mode, grid	No	Static	Single robot, automatic container terminal	Yes	Simulation
[59]	ACO, GWO	Path smoothness, convergence	Grid	No	Static	Single robot	Yes	Simulation
[69]	ACO, DWA	Turns, path length	Grid	No	Static	Single robot, indoor environment	Yes	Simulation, Experiment
[70]	ACO	Material flow and path length	Raster map	No	Static	Single robot, job shop	No	Simulation
[71]	ACO, GA	Distance, iterations	Grid map	No	Static	Single robot	No	Simulation
[72]	ACO	Distance factors, task execution time, waiting time	Grid map	No	Static	Multi-robots, factory environment	No	Simulation

**Table 3:** Comparison of the GA-based algorithms.

Paper	Algorithm	Consideration	Model	Online	Properties	Scenario	Hybrid	Experiment
[73]	GA, Dijkstra, time window	Minimize the make span, the number of AGVs	Grid space	No	Static	Multi-robots, flexible manufacturing system	Yes	Simulation
[74]	GA, PSO, fuzzy logic controller	Delayed completion time, deadlocks	Grid space	No	Static	Multi-robots, automated container terminals	Yes	Simulation
[76]	GA, heuristic	Intercellular transportation and makespan-related costs	Grid space	No	Static	Multi-robots, cellular manufacturing system	Yes	Simulation
[77]	GA, EDA	Flight heights, blocking of buildings	Grid space	Yes	Dynamic	Multi-robots, cooperative, surveillance, urban environment	Yes	Simulation
[78]	GA, SA	Path smoothness	Grid	No	Static	Single robot	Yes	Simulation, Experiment
[79]	GA	Smooth and safe movement	Grid	No	Static	Multi-robots, Cooperative	No	Simulation, Experiment
[80]	GA, A*	Task completion time, energy consumption	Raster map, Grid	Yes	Static	Multi-robots	Yes	Simulation
[75]	GA	Completion time	Road network model	No	Static	Multi-robots	Yes	Simulation
[81]	GA	Path length	2D map	Yes	Dynamic	Multi-robots	No	Simulation

**Table 4:** Comparison of the NN-based models.

Paper	Algorithm	Consideration	Model	Online	Properties	Scenario	Hybrid	Experiment
[82]	Neural network, the Bellman–Ford algorithm, a quadratic program	The sum of the distance	Grid-based graph	Yes	Static	Single robot	Yes	Simulation
[83]	RDNN, LSTM	Collision, time, process and terminal costs	-	Yes	Static	Single robot, parking	Yes	Simulation, Experiment
[84]	Neural network, ACO	Path length	Grid	Yes	Static	Single robot	Yes	Simulation
[85]	NAR neural network, A*	Velocity, motion path	2D map	Yes	Dynamic	Moving single target	Yes	Simulation
[44]	Deep neural network (DNN)	Path length, target, obstacles	Grid	Yes	Dynamic	Single robot	Yes	Simulation

**Table 5:** Comparison of the RL-based models.

Paper	Algorithm	Consideration	Model	Online	Properties	Scenario	Hybrid	Experiment
[91]	DRL, RNN, PPO, LSTM	Position, obstacles, distance, spacing	Grid	Yes	Dynamic	Multiple robots, automated storage and retrieval system (AS/RS)	Yes	Simulation
[97]	Q-Learning	Path length and smoothness	Graph	Yes	Static	Single robot	No	Simulation, Experiment
[102]	Q-learning	Collision, terminal state	Grid	No	Static	Multiple robots	Yes	Simulation
[100]	Q-learning	Distance	Grid	No	Static	Multiple robots	Yes	Simulation
[105]	Q-learning	Turning rewards, dynamic priority, action replanning	Grid	No	Static	Multiple robots	Yes	Simulation
[98]	Q-learning	Convergence, path length	Grid	Yes	Dynamic	Multiple robots	Yes	Simulation, Experiment
[99]	Q-learning	Target, obstacles	Grid	No	Static	Single robot	No	Simulation
[101]	Q-learning	Locations, destinations	Grid	Yes	Dynamic	Multiple robots, production logistics system	Yes	Simulation, Experiment
[104]	Q-learning, ACO, GA	Distance, congestion time, charging priority	Grid	No	Static	Single robot, shared charging system	Yes	Simulation
[103]	Q-learning, beetle antennae search (BAS)	Path length, average time	Grid	No	Static	Single robot	Yes	Simulation
[106]	Deep Q-learning	Obstacles, target	Grid	No	Static	Single robot, intelligent manufacturing workshops	Yes	Simulation
[95]	PPO, LSTM	Distance, heading angle, collision, target point	Grid	Yes	Dynamic	Single robot	Yes	Simulation, Experiment
[93]	PPO	Static and dynamic obstacles	Grid	Yes	Dynamic	Single robot	Yes	Simulation
[92]	PPO, LSTM	Distance, collision	Grid	Yes	Dynamic	Single robot	Yes	Simulation
[94]	MAPPO, GNN	Position, velocity, obstacle	Grid	Yes	Dynamic	Multiple robots	Yes	Simulation
[96]	MAPPO	Movement, obstacles, global path, target, boundary	Grid	Yes	Dynamic	Single robot	Yes	Simulation
[107]	DDPG, APF	Smoothness and safety	Graph	No	Static	Single robot	Yes	Simulation
[111]	DRL	Collision, movement, finish task	Grid	Yes	Static	Multiple robots	Yes	Simulation
[112]	Dyna-Q	Goal	Grid	Yes	Static	Single robot	Yes	Simulation
[110]	Dyna-Q, ACO	Obstacle, target	Grid	Yes	Dynamic	Single robot	Yes	Simulation
[109]	SAC	Obstacle, distance, target and time	Grid	Yes	Dynamic	Single robot	Yes	Simulation
[108]	MADDPG	Position, collision, speed	Grid	No	Static	Multiple robots	Yes	Simulation
[90]	D3QN, A*	Average tardiness and energy consumption	Grid	Yes	Dynamic	Multiple robots	Yes	Simulation
[88]	DQN	Direction, steps, end point	Grid	Yes	Dynamic	Single robot	Yes	Simulation
[89]	Dueling DQN	Position, velocity, target	Grid	Yes	Dynamic	Single robot, intelligent logistics systems	Yes	Simulation

**Table 6:** Comparison of the FL-based models.

Paper	Algorithm	Consideration	Model	Online	Properties	Scenario	Hybrid	Experiment
[113]	Fuzzy logic, ACO	Pollutant emissions, fuel cost, travel time, and distance	Grid	Yes	Dynamic	Single robot	Yes	Simulation
[114]	Fuzzy logic, APF	Obstacles, velocities, lane lines	2D map	Yes	Dynamic	Single robot, automated terminals, port	Yes	Simulation
[115]	Fuzzy control, ACO, DWA	Safety, smoothness, distance, direction	Grid	Yes	Dynamic	Single robot	Yes	Simulation
[116]	Fuzzy control, A*, DWA	Path length, path search, smoothness	Grid	Yes	Static	Single robot	Yes	Simulation

**Table 7:** Comparison of other algorithms.

Paper	Classification	Algorithm	Consideration	Model	Online	Properties	Scenario	Hybrid	Experiment
[118]	Swarm intelligence	Jaya	Minimize transportation cost, total tardiness, early service penalty	Grid space	No	Static	Multi-robots, matrix manufacturing workshop	No	Simulation
[123]	Swarm intelligence	Artificial fish swarm algorithm	Safety, fuel economy, trajectory smoothness	Grid space	Yes	Dynamic	Single robot	Yes	Simulation, Experiment
[119]	Swarm intelligence	Fireworks algorithm, APF	Safety, path smoothness	-	Yes	Dynamic	Single robot, driving	Yes	Simulation, Experiment
[120]	Swarm intelligence	Sparrow search algorithm	Risk degree, path acquisition time, distance value, total rotation angle value	Grid space	No	Static	Single robot	No	Simulation
[121]	Swarm intelligence	GWO, Kalman filter	Path smoothness and length, obstacle avoidance	Grid space	No	Static	Single robot	Yes	Simulation
[122]	Swarm intelligence	DE	Make span, collision	Workshop diagram	No	Static	Multi-robots	Yes	Simulation
[117]	Artificial intelligence	SVM	Path length	Grid	No	Static	Multiple robots	Yes	Simulation

**Table 8:** Comparison of swarm intelligence algorithms.

Paper	Algorithm	Contribution	Limitation/Future Research
[2]	MOPSO	Formulate energy-efficient AGV path planning model, two solution methods	Energy consumption data acquisition, integration of transport task execution, multi-AGV system
[49]	MOPSO, DWA	Combines MOPSO and DWA for optimization challenges and dynamics	Environmental uncertainties, changing environmental conditions, real-world experiments
[46]	PSO	Crossover operation, mutation mechanism, local optimum problem	Multi-AGV system
[5]	PSO, SA	Get rid of local optima, accept new solution, and update local-oriented best value with a probability	Dynamic environment, multiple robots, moving obstacles
[48]	PSO, GWO	Local search technique	Not multi-objective optimization, real-time implementation, multi-robots, moving goal
[50]	PSO	Alpha and beta as two coefficients	Path prediction and learning capabilities, only static simple environment
[52]	PSO	Levy flight, inductive steering algorithm	Dynamic situation is simple
[53]	PSO, ACO	A collision avoidance factor, avoid road-section and node conflicts	Only static environment
[47]	PSO, CA	The cultural-PSO algorithm, dynamic adjust inertial weight	Real-world experiment
[54]	PSO, ACO	PSO-IACO, PSO optimizes initial parameters of ACO	Only static environment, lack of real-world experiment
[51]	PSO, Human optimization algorithm	PSO combines HLO	Multi robots, dynamic environment
[55]	ACO	Heuristic information, compare the similarity of the job, path planning and scheduling	Limited robustness, other manufacturing environments (flexible job-shop or flow shop)
[56]	ACO	Fallback strategy, valuation function, reward/penalty mechanism	The efficiency of the algorithm
[57]	ACO	Penalty strategy	Multiple robots, experiment
[58]	ACO	Initial pheromone concentration, improved state transition probability rule	Three-dimensional problem, multi-objective optimization, execution time
[60]	ACO, Dijkstra	ACO-DA	Multi-AGV conflicts
[61]	ACO, A* Multi-Directional algorithm	Reward policy	Dynamic moving obstacles
[62]	ACO, rolling window	The pheromone concentration	Optimization, convergence performance, the scope of application
[63]	ACO, RRT*	Fast-scaling RRT*-ACO	Only static environment
[64]	ACO	Step length, adaptive pheromone volatilization coefficient	Multi-AGVs’ conflict resolution
[65]	ACO	Hexagonal grid map model, the bidirectional search strategy	Global search optimization, grid map’s robustness, real-world application, efficiency
[66]	ACO	RL configures ACO parameters	Lack comparison analysis
[67]	ACO	Bloch coordinates of pheromones; a repulsion factor	Uncertain environments, task assignment, real automated logistics systems
[68]	ACO	Combines FOA and ACO	Lack comparison analysis
[59]	ACO, GWO	A modified ACO based on GWO, heuristic information, the pheromone model, and transfer rules	Only static environment, lack comparison analysis
[69]	ACO, DWA	Combine ACO and DWA	Focus on global path planning, and the static environment is not complex
[70]	ACO	Additional heuristic information, dynamic adjustment factor, Laplace distribution	Dynamic simulation and scheduling
[71]	ACO, GA	Non-uniform and directed distribution of initial pheromone, adaptive adjustment, parameter optimization by GA	Lack comparison
[72]	ACO	Prior time, the pheromone increment	Large-scale and changing tasks
[73]	GA, Dijkstra, time window	Global, local and random search strategies, optimize the number of AGVs	Dynamic scheduling and job sequencing problem
[74]	GA, PSO, fuzzy logic controller	Integrated scheduling and path planning, adaptive auto tuning	Computation time, dynamic real-time scheduling
[76]	GA, heuristic	Applying the fuzzy linear programming, hybrid approach	Complicate AGVs’ constraints, not real case
[77]	GA, EDA	Cooperative path planning model, online adjustment strategy	More possible applications
[78]	GA, SA	Path smoothness constraints, crossover stage, mutation operation	Lacks comparison with state-of-the-art techniques
[79]	GA	Fitness function	Only consider static obstacles
[80]	GA, A*	A* combines cyclic rules, GA with penalty function	Only static obstacle, AGV charging problem in the future
[75]	GA	A three-stage optimal scheduling algorithm	Lacks comparison analysis, AGV charging, collision avoidance route
[81]	GA	Improved GA, two decision variables	Lacks comparison analysis
[118]	Jaya	The key-task shift method, initialization methods, offspring generation methods, insertion-based repair method	Considers more practical constraints and production environments, the use of multi-objective optimization problem and new techniques
[123]	Artificial fish swarm algorithm	Trail-based forward search algorithm, command signals	Lacks comparison with state-of-the-art techniques
[119]	Fireworks algorithm, APF	DynEFWA-APF	Incorporates personalized driving style
[120]	Sparrow search algorithm	Location update formula, neighborhood search strategy, linear path strategy	Experiment, multi-robots, dynamic obstacles
[121]	GWO, Kalman filter	Refine with KF corrections	Only static environment
[122]	DE	Hybrid variable neighborhood DE	AGVs’ speed, multi-objective optimization

**Table 9:** Comparison of artificial intelligence algorithms.

Paper	Algorithm	Contribution	Limitation/Future Research
[82]	Neural network, the Bellman–Ford algorithm, a quadratic program	Offline training, and online path planning	Hard to acquire perfect situational awareness, trained data, dimensionality
[83]	RDNN, LSTM	RNDD-based motion planning, transfer learning strategies	Multi-robot environment
[84]	Neural network, ACO	Combines ACO with neural networks	The environmental model is not clear
[85]	NAR neural network, A*	Reduced and non-reduced point	The success rate is fair
[44]	Deep neural network (DNN),	Target area adaptive RRT*, optimal path backward generation, DNN	Consider kinematic information, 3D scenarios, and transfer learning in future studies
[113]	Fuzzy logic, ACO	FLACO, local optimum trap, global optimal path	Reducing the computing time, multiple vehicles
[114]	Fuzzy logic, APF	Hybrid APF-fuzzy model prediction controller	AGV modeling
[115]	Fuzzy control, ACO, DWA	Improved ACO and DWA with fuzzy controllers	Only static obstacles
[116]	Fuzzy control, A*, DWA	Adapative neuro-fuzzy inference system, enhanced A* with DWA	Robustness, applicability, real-world environments
[87]	RL DQN, A*	Slow convergence and excessive randomness	Local path planning
[86]	DQN	State-dynamic network model	Multi-AGV environment
[91]	DRL, RNN, PPO, LSTM	Temporary changes	Reduce the computational time, dynamic conflict avoidance strategies
[97]	Q-Learning	Global Q-learning path planning	Lack the modification of Q-learning
[102]	Q-learning	Behavior trees	Not considering completed situations, AGV scheduling, or real-world system
[100]	Q-learning	Contract net protocol	The comparison analysis is weak; it only uses traditional Q-learning
[105]	Q-learning	Map training and action replanning	Dynamics of AGVs are not considered; the environment is simple
[98]	Q-learning	Kohonen Q-learning	Task scheduling and assignment
[99]	Q-learning	A deep learning factor	Static obstacle environment
[101]	Q-learning	Digital Twin-driven Q-learning	More complex situations, task allocation
[104]	Q-learning, ACO, GA	Q-learning and ACO, positive ant colony feedback mechanism	Only compared with Dijkstra and A* algorithm, static environment
[103]	Q-learning, beetle antennae search (BAS)	BAS-QL	Static obstacles
[106]	Deep Q-learning	Experience replay pool, network structure, neighborhood weighted grid modeling	Dynamic environments should be studied
[95]	PPO, LSTM	Introduce ICM and LSTM into PPO	The success rate decreases when dynamic obstacles moving fast or not follow regular patterns
[93]	PPO	Additional intrinsic rewards	Cannot guarantee safety in the training
[92]	PPO, LSTM	Sample regularization, adaptive learning rate	Lack environmental experiments
[94]	MAPPO, GNN	GNN with MADRL	Complex interactions and dynamic environment
[96]	MAPPO	A* for global guidance, MAPPO for local planning	Multi-robot scenario
[107]	DDPG, APF	APF, twin delayed DDPG	Lack environmental perception and testing, real experiment, and hard to implement in complex environment
[111]	DRL	Local observations	High density of obstacles
[112]	Dyna-Q	Heuristic planning	Lacks comparison with SOTA methods
[110]	Dyna-Q, ACO	Improved heuristic function of ACO, combines with Dyna-Q	Lacks comparison analysis
[109]	SAC	Sum-tree replay	Lacks experiments
[108]	MADDPG	*ϵ*-Greedy	Optimal value has not been established
[90]	D3QN, A*	Digital twin, prevent deadlock and congestion	Multi-resource production scheduling problems
[88]	DQN	A refined multi-objective reward function, the priority experience replay mechanism	Robust training methods, dynamic obstacle prediction modules, experimental design
[89]	Dueling DQN	Multimodal sensing information, prioritized experience reply	MARL
[117]	SVM	SVM-based model, replanning period	Model transfer methodology

## Data Availability

No new data were created or analyzed in this study.

## Abbreviations

The following abbreviations are used in this manuscript:

AGV	Automated guided vehicle
RRT	Rapidly-exploring random tree
APF	Artificial potential field
PRM	Probabilistic roadmap
PSO	Particle swarm optimization
SA	Simulated annealing
GWO	Grey wolf optimizer
MOPSO	Multi-objective particle swarm optimization
DWA	Dynamic window approach
FOA	Fruit fly optimization algorithm
EDA	Estimation of distribution algorithm
LSTM	Long short-term memory
DQN	Deep-Q network
D3QN	Dueling double deep-Q network
DRL	Deep reinforcement learning
RNN	Recurrent neural network
PPO	Proximal policy optimization
ICM	Intrinsic curiosity module
DDPG	Deep deterministic policy gradient
MPC	Model predictive controller
DE	Differential evolution
FL	Fuzzy logic
